# *N*-(1,3-Thia­zol-2-yl)benzamide

**DOI:** 10.1107/S1600536809009374

**Published:** 2009-03-25

**Authors:** Afsaneh Zonouzi, Roghieh Mirzazadeh, Hossein Rahmani, Seik Weng Ng

**Affiliations:** a Department of Chemistry, College of Science, University of Tehran, PO Box 13145-143, Tehran, Iran; b Institute of Chemical Industries, Iranian Research Organization for Science and Technology, PO Box 15815-358, Tehran, Iran; c Department of Chemistry, University of Malaya, 50603 Kuala Lumpur, Malaysia

## Abstract

The title compound, C_10_H_8_N_2_OS, features a nonplanar mol­ecule [dihedral angle between the two aromatic rings = 43.6 (1)°]. Two mol­ecules are linked by N—H⋯N hydrogen bonds about a centre of inversion, giving rise to a hydrogen-bonded dimer.

## Related literature

The synthesis uses microwave radiation, which compares with benzoyl­ation by reacting benzoyl cyanide in an ionic liquid: see: Kumar *et al.* (2007 ▶); Prasad *et al.* (2005 ▶).
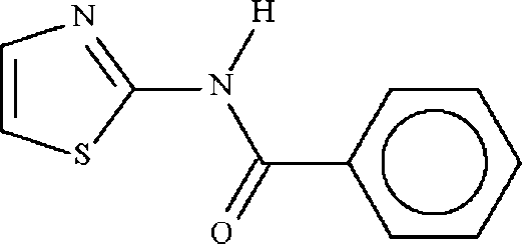


## Experimental

### 

#### Crystal data

C_10_H_8_N_2_OS
*M* *_r_* = 204.24
Monoclinic, 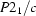

*a* = 12.0142 (2) Å
*b* = 5.0581 (1) Å
*c* = 15.4090 (3) Å
β = 99.093 (1)°
*V* = 924.62 (3) Å^3^
*Z* = 4
Mo *K*α radiation
μ = 0.31 mm^−1^
*T* = 123 K
0.35 × 0.20 × 0.15 mm

#### Data collection

Bruker SMART APEXdiffractometer
Absorption correction: multi-scan (*SADABS*; Sheldrick, 1996 ▶) *T*_min_ = 0.898, *T*_max_ = 0.955
6130 measured reflections
2104 independent reflections
1900 reflections with *I* > 2σ(*I*)
*R*_int_ = 0.016

#### Refinement

*R*[*F* ^2^ > 2σ(*F* ^2^)] = 0.029
*wR*(*F* ^2^) = 0.087
*S* = 1.07
2104 reflections
131 parameters
H atoms treated by a mixture of independent and constrained refinement
Δρ_max_ = 0.37 e Å^−3^
Δρ_min_ = −0.21 e Å^−3^

### 

Data collection: *APEX2* (Bruker, 2008 ▶); cell refinement: *SAINT* (Bruker, 2008 ▶); data reduction: *SAINT*; program(s) used to solve structure: *SHELXS97* (Sheldrick, 2008 ▶); program(s) used to refine structure: *SHELXL97* (Sheldrick, 2008 ▶); molecular graphics: *X-SEED* (Barbour, 2001 ▶); software used to prepare material for publication: *publCIF* (Westrip, 2009 ▶).

## Supplementary Material

Crystal structure: contains datablocks global, I. DOI: 10.1107/S1600536809009374/bt2897sup1.cif

Structure factors: contains datablocks I. DOI: 10.1107/S1600536809009374/bt2897Isup2.hkl

Additional supplementary materials:  crystallographic information; 3D view; checkCIF report

## Figures and Tables

**Table 1:** Hydrogen-bond geometry (Å, °)

*D*—H⋯*A*	*D*—H	H⋯*A*	*D*⋯*A*	*D*—H⋯*A*
N2—H2⋯N1^i^	0.88 (2)	2.04 (2)	2.922 (2)	173 (2)

Symmetry code: (i) 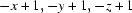.

**Fig. 1.**
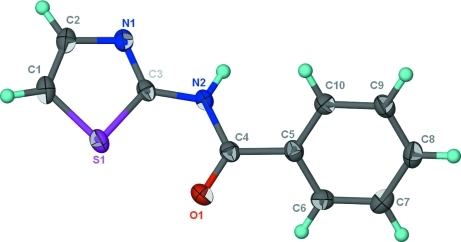
Anisotropic displacement ellisoid plot (Barbour, 2001) of C10H8N2OS; probability levels are set at 70% and H-atoms are drawn as spheres of arbitrary radius.

## supplementary crystallographic information

### Experimental

2-Aminothiazole (1 g, 10 mmol) and benzoyl cyanide (1.31 g, 10 mmol) were stirred together without any solvent for 3 h at 323 K. The oily product was purified by recrystalization from ethanol (yield 1.97 g, 90%); m.p. 383 K.

### Refinement

Carbon-bound H-atoms were placed in calculated positions (C–H 0.95 Å) and were included in the refinement in the riding model approximation, with *U*(H) set to 1.2*U*(C).

The amino H-atom was located in a difference Fouier map, and was freely refined.

### Crystal data

**Table:**

C_10_H_8_N_2_OS	*F*(000) = 424
*M**_r_* = 204.24	*D*_x_ = 1.467 Mg m^−^^3^
Monoclinic, *P*2_1_/*c*	Mo *K*α radiation, λ = 0.71073 Å
Hall symbol: -P 2ybc	Cell parameters from 3661 reflections
*a* = 12.0142 (2) Å	θ = 2.7–28.3°
*b* = 5.0581 (1) Å	µ = 0.31 mm^−^^1^
*c* = 15.4090 (3) Å	*T* = 123 K
β = 99.093 (1)°	Prism, colorless
*V* = 924.62 (3) Å^3^	0.35 × 0.20 × 0.15 mm
*Z* = 4	

### Data collection

**Table:**

Bruker SMART APEX diffractometer	2104 independent reflections
Radiation source: fine-focus sealed tube	1900 reflections with *I* > 2σ(*I*)
graphite	*R*_int_ = 0.016
ω scans	θ_max_ = 27.5°, θ_min_ = 1.7°
Absorption correction: multi-scan (*SADABS*; Sheldrick, 1996)	*h* = −15→15
*T*_min_ = 0.898, *T*_max_ = 0.955	*k* = −6→6
6130 measured reflections	*l* = −18→20

### Refinement

**Table:**

Refinement on *F*^2^	Primary atom site location: structure-invariant direct methods
Least-squares matrix: full	Secondary atom site location: difference Fourier map
*R*[*F*^2^ > 2σ(*F*^2^)] = 0.029	Hydrogen site location: inferred from neighbouring sites
*wR*(*F*^2^) = 0.087	H atoms treated by a mixture of independent and constrained refinement
*S* = 1.07	*w* = 1/[σ^2^(*F*_o_^2^) + (0.0497*P*)^2^ + 0.3231*P*] where *P* = (*F*_o_^2^ + 2*F*_c_^2^)/3
2104 reflections	(Δ/σ)_max_ = 0.001
131 parameters	Δρ_max_ = 0.37 e Å^−^^3^
0 restraints	Δρ_min_ = −0.21 e Å^−^^3^

### Fractional atomic coordinates and isotropic or equivalent isotropic displacement parameters (Å^2^)

**Table:**

	*x*	*y*	*z*	*U*_iso_*/*U*_eq_	
S1	0.31389 (3)	0.14766 (7)	0.64434 (2)	0.02139 (12)	
O1	0.17527 (8)	0.5276 (2)	0.56442 (6)	0.0229 (2)	
N1	0.49298 (9)	0.2290 (2)	0.57510 (7)	0.0196 (2)	
N2	0.34258 (9)	0.4997 (2)	0.51416 (7)	0.0186 (2)	
H2	0.3903 (16)	0.574 (4)	0.4833 (13)	0.039 (5)*	
C1	0.43025 (12)	−0.0499 (3)	0.67627 (9)	0.0229 (3)	
H1	0.4338	−0.1892	0.7180	0.028*	
C2	0.51534 (11)	0.0214 (3)	0.63371 (8)	0.0210 (3)	
H2A	0.5863	−0.0656	0.6434	0.025*	
C3	0.38904 (11)	0.3090 (2)	0.57304 (8)	0.0173 (3)	
C4	0.23427 (11)	0.5903 (3)	0.50994 (8)	0.0179 (3)	
C5	0.19345 (10)	0.7638 (3)	0.43320 (8)	0.0178 (3)	
C6	0.11908 (11)	0.9675 (3)	0.44382 (9)	0.0207 (3)	
H6	0.0981	0.9989	0.4998	0.025*	
C7	0.07532 (11)	1.1252 (3)	0.37287 (9)	0.0239 (3)	
H7	0.0251	1.2655	0.3804	0.029*	
C8	0.10522 (11)	1.0769 (3)	0.29083 (9)	0.0235 (3)	
H8	0.0754	1.1848	0.2422	0.028*	
C9	0.17835 (11)	0.8722 (3)	0.27946 (9)	0.0221 (3)	
H9	0.1978	0.8389	0.2231	0.026*	
C10	0.22320 (11)	0.7157 (3)	0.35072 (8)	0.0199 (3)	
H10	0.2739	0.5765	0.3432	0.024*	

### Atomic displacement parameters (Å^2^)

**Table:**

	*U*^11^	*U*^22^	*U*^33^	*U*^12^	*U*^13^	*U*^23^
S1	0.02421 (19)	0.02313 (19)	0.01715 (19)	−0.00215 (12)	0.00419 (13)	0.00464 (12)
O1	0.0245 (5)	0.0273 (5)	0.0181 (5)	0.0007 (4)	0.0071 (4)	0.0020 (4)
N1	0.0231 (5)	0.0187 (5)	0.0170 (5)	0.0014 (4)	0.0027 (4)	0.0016 (4)
N2	0.0201 (5)	0.0202 (5)	0.0160 (5)	0.0008 (4)	0.0049 (4)	0.0042 (4)
C1	0.0300 (7)	0.0190 (6)	0.0181 (6)	−0.0018 (5)	−0.0012 (5)	0.0027 (5)
C2	0.0260 (6)	0.0174 (6)	0.0182 (6)	0.0013 (5)	−0.0010 (5)	0.0002 (5)
C3	0.0223 (6)	0.0170 (6)	0.0125 (6)	−0.0019 (5)	0.0027 (5)	−0.0008 (4)
C4	0.0210 (6)	0.0184 (6)	0.0143 (6)	−0.0004 (5)	0.0028 (5)	−0.0018 (5)
C5	0.0180 (6)	0.0189 (6)	0.0161 (6)	−0.0021 (5)	0.0015 (5)	0.0008 (5)
C6	0.0181 (6)	0.0242 (6)	0.0201 (6)	−0.0001 (5)	0.0038 (5)	−0.0028 (5)
C7	0.0206 (6)	0.0209 (6)	0.0291 (7)	0.0022 (5)	0.0007 (5)	−0.0006 (5)
C8	0.0213 (6)	0.0241 (6)	0.0231 (7)	−0.0013 (5)	−0.0022 (5)	0.0061 (5)
C9	0.0221 (6)	0.0277 (7)	0.0163 (6)	−0.0026 (5)	0.0028 (5)	0.0017 (5)
C10	0.0200 (6)	0.0217 (6)	0.0180 (6)	0.0015 (5)	0.0032 (5)	0.0001 (5)

### Geometric parameters (Å, °)

**Table:**

S1—C1	1.7255 (14)	C5—C6	1.3903 (18)
S1—C3	1.7327 (13)	C5—C10	1.3949 (18)
O1—C4	1.2231 (16)	C6—C7	1.3877 (19)
N1—C3	1.3084 (17)	C6—H6	0.9500
N1—C2	1.3834 (16)	C7—C8	1.389 (2)
N2—C4	1.3714 (17)	C7—H7	0.9500
N2—C3	1.3801 (16)	C8—C9	1.387 (2)
N2—H2	0.88 (2)	C8—H8	0.9500
C1—C2	1.348 (2)	C9—C10	1.3913 (18)
C1—H1	0.9500	C9—H9	0.9500
C2—H2A	0.9500	C10—H10	0.9500
C4—C5	1.4919 (17)		
			
C1—S1—C3	88.49 (6)	C6—C5—C4	118.65 (11)
C3—N1—C2	109.69 (11)	C10—C5—C4	121.33 (12)
C4—N2—C3	123.16 (11)	C7—C6—C5	120.24 (12)
C4—N2—H2	121.6 (13)	C7—C6—H6	119.9
C3—N2—H2	114.8 (13)	C5—C6—H6	119.9
C2—C1—S1	110.43 (10)	C6—C7—C8	119.71 (13)
C2—C1—H1	124.8	C6—C7—H7	120.1
S1—C1—H1	124.8	C8—C7—H7	120.1
C1—C2—N1	115.88 (12)	C7—C8—C9	120.40 (12)
C1—C2—H2A	122.1	C7—C8—H8	119.8
N1—C2—H2A	122.1	C9—C8—H8	119.8
N1—C3—N2	121.17 (11)	C8—C9—C10	119.95 (13)
N1—C3—S1	115.46 (10)	C8—C9—H9	120.0
N2—C3—S1	123.29 (10)	C10—C9—H9	120.0
O1—C4—N2	121.95 (12)	C5—C10—C9	119.78 (12)
O1—C4—C5	122.90 (12)	C5—C10—H10	120.1
N2—C4—C5	115.14 (11)	C9—C10—H10	120.1
C6—C5—C10	119.91 (12)		
			
C3—S1—C1—C2	1.28 (10)	N2—C4—C5—C6	−146.32 (12)
S1—C1—C2—N1	−0.31 (15)	O1—C4—C5—C10	−141.00 (14)
C3—N1—C2—C1	−1.25 (16)	N2—C4—C5—C10	37.46 (17)
C2—N1—C3—N2	−174.45 (11)	C10—C5—C6—C7	−0.75 (19)
C2—N1—C3—S1	2.29 (14)	C4—C5—C6—C7	−177.03 (11)
C4—N2—C3—N1	−179.70 (12)	C5—C6—C7—C8	0.7 (2)
C4—N2—C3—S1	3.83 (17)	C6—C7—C8—C9	0.1 (2)
C1—S1—C3—N1	−2.12 (10)	C7—C8—C9—C10	−0.7 (2)
C1—S1—C3—N2	174.53 (11)	C6—C5—C10—C9	0.09 (19)
C3—N2—C4—O1	7.78 (19)	C4—C5—C10—C9	176.27 (12)
C3—N2—C4—C5	−170.70 (11)	C8—C9—C10—C5	0.6 (2)
O1—C4—C5—C6	35.22 (18)		

### Hydrogen-bond geometry (Å, °)

**Table:**

*D*—H···*A*	*D*—H	H···*A*	*D*···*A*	*D*—H···*A*
N2—H2···N1^i^	0.88 (2)	2.04 (2)	2.922 (2)	173 (2)

Symmetry codes: (i) −*x*+1, −*y*+1, −*z*+1.
